# Direct evidence of Neanderthal fibre technology and its cognitive and behavioral implications

**DOI:** 10.1038/s41598-020-61839-w

**Published:** 2020-04-09

**Authors:** B. L. Hardy, M.-H. Moncel, C. Kerfant, M. Lebon, L. Bellot-Gurlet, N. Mélard

**Affiliations:** 1 Department of Anthropology, Kenyon College, Gambier, Ohio USA; 2 Histoire Naturelle de l’Homme Préhistorique (HNHP), UMR 7194, Dept. Homme et Environnement du Muséum national d’Histoire Naturelle, CNRS, UPVD, Institut de Paleontologie Humaine-Musée de l’Homme, 75013, Paris, France; 3 Àrea de Prehistòria, Universitat Rovira i Virgili (URV), Av. Catalunya 35, 43002 Tarragona, Spain; 4 IPHES, Institut Català de Paleoecologia Humana i Evolució Social, Zona Educacional 4 Campus Sescelades URV (Edifici W3), 43007 Tarragona, Spain; 5 5 Sorbonne Université, CNRS, De la Molécule aux Nano-objets: Réactivité, Interactions et Spectroscopies (MONARIS), UMR 8233, 75005 Paris, France; 6 Centre de Recherche et de Restauration des Musées de France, C2MRF Palais du Louvre, Paris, France

**Keywords:** Palaeoecology, Archaeology

## Abstract

Neanderthals are often considered as less technologically advanced than modern humans. However, we typically only find faunal remains or stone tools at Paleolithic sites. Perishable materials, comprising the vast majority of material culture items, are typically missing. Individual twisted fibres on stone tools from the Abri du Maras led to the hypothesis of Neanderthal string production in the past, but conclusive evidence was lacking. Here we show direct evidence of fibre technology in the form of a 3-ply cord fragment made from inner bark fibres on a stone tool recovered *in situ* from the same site. Twisted fibres provide the basis for clothing, rope, bags, nets, mats, boats, etc. which, once discovered, would have become an indispensable part of daily life. Understanding and use of twisted fibres implies the use of complex multi-component technology as well as a mathematical understanding of pairs, sets, and numbers. Added to recent evidence of birch bark tar, art, and shell beads, the idea that Neanderthals were cognitively inferior to modern humans is becoming increasingly untenable.

## Introduction

**Figure 1:**
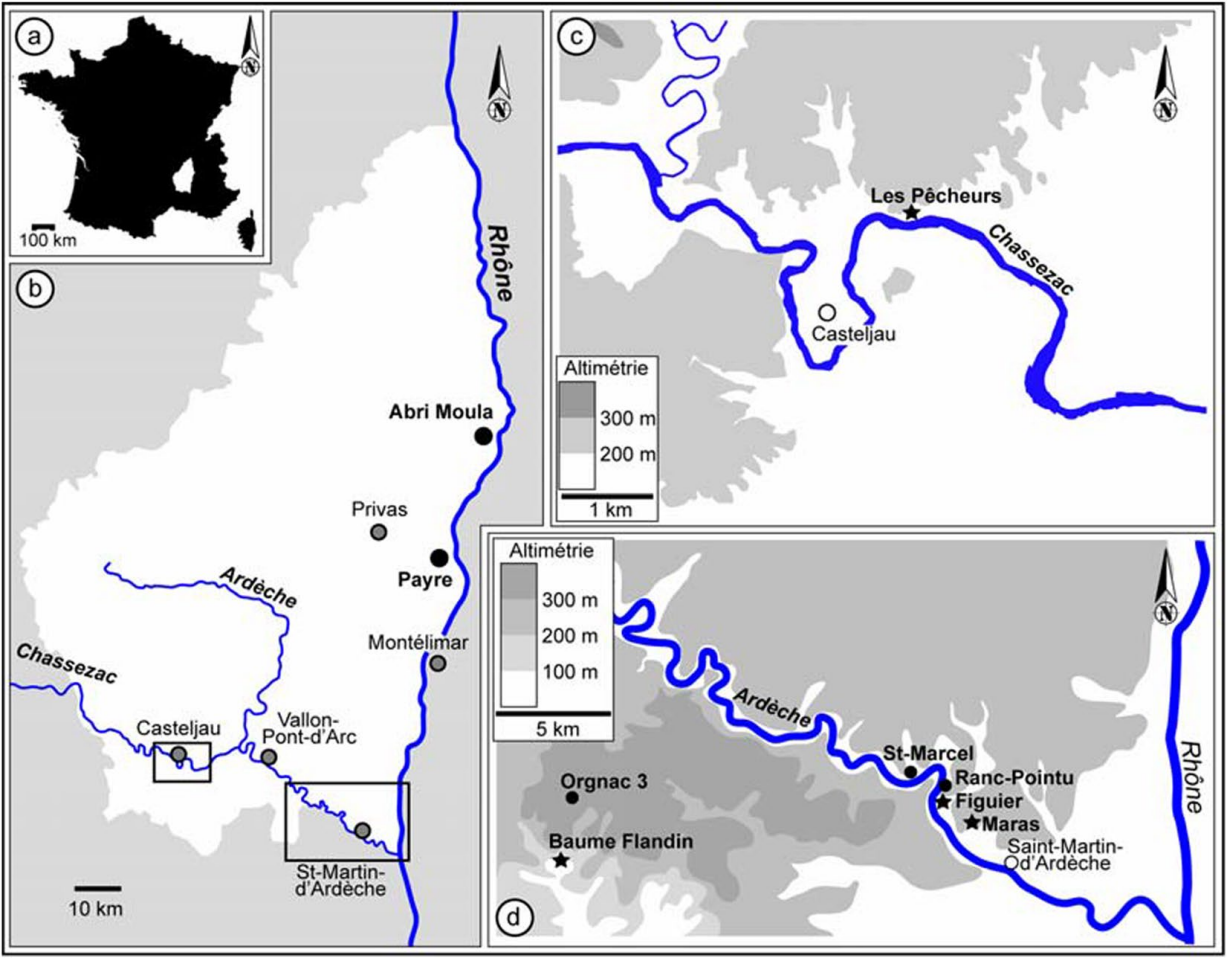
Map showing location of Abri du Maras. (Map by S. Puaud).

With a few exceptions such as the Schöningen spears^1^ and the recent finds of wooden tools at Pogetti Vecchi^2^, almost all of our knowledge about the Middle Paleolithic comes from durable materials (bones and stone tools)^3,4^. We know from observations of our own surroundings, ethnographic and ethnohistoric accounts that most of the material culture of humans (and Neanderthals) is comprised of perishable materials^5^. Hurcombe^4^ has called this problem “the missing majority”. Obviously, differential preservation of materials contributes to this bias. Previously, researchers have demonstrated that the microenvironment immediately surrounding a stone tool can preserve microscopic fragments of what is otherwise invisible archaeologically^6,7^. This is also true for the preservation of a 3-ply cordage fragment adhering to a stone tool (flake) from Abri du Maras (Fig. 1).

The Abri du Maras is located in a valley near the Ardèche River, a tributary of the Rhône River (Fig. 1). New excavations have taken place since 2006 and focus on the Middle Paleolithic (MP) occupations. This site had previously yielded MP deposits with Levallois laminar debitage at the top of the sequence^8,9^.

The oldest occupations took place under a large cave roof, which collapsed over time, and the youngest occupations were under a rockshelter. Two main layers with distinct levels of occupation were discovered (Units 5 and 4) during the new excavations located among beds of limestone blocks. The site has been dated by ESR and U-Th methods. Unit 5 is dated to the end of the MIS 5/beginning of MIS 4 at 90 thousand years (ka). Level 4.1 is dated to between 40 ± 3 ka and 46 ± 3 ka (MIS 3), while samples from Level 4.2 provided ages ranging from 41 ± 2 ka, 46 ± 5 ka to 52 ± 2 ka (MIS 3)^10^. The cordage remains are from Level 4.2.

Unit 4 comprises a 0.5–1 m thick layer of silt and sandy-silt located 3 m below the modern surface. Frost-shattering of the rock shelter walls produced the coarse fraction while the fine loess-like sediment is of aeolian origin. Geological investigations indicate that most of the deposit originated during MIS 3 due to wind erosion of fluvio-glacial terraces in the region^11^. Micromorphology indicates that unit 4 has undergone moderate pedological evolution in the form of biogenic redistribution of calcium carbonate. The clayey-sandy silt sedimentary matrix associated with the archaeological remains is similar in color, structure and texture throughout.

Unit 4, including more than 50 m^2^ of excavation, contains two archaeological levels divided by a sterile deposit. These two levels (4.2 and 4.1) represent two human occupation phases with abundant artefacts and some traces of combustion and diffuse ash lenses. The overlying units (3, 2, and 1) are coarse in texture and contain large limestone blocks. These units have yielded only a few scattered artefacts.

From a paleoclimatic perspective, layer 4 was deposited during progressively colder and drier conditions with a majority of the fauna being reindeer (*Rangifer tarandus*)^12^. More than 4,000 artefacts longer than 15 mm have been found in Unit 4 (40 to 50 artefacts/m^3^). The flaking is mainly of Levallois type, associated with other core technologies, with an assemblage of mostly unretouched flint flakes, blades, bladelets and points made on local flint (collected within 30 km of the site)^13^. Large flakes, blades and points are brought already worked from outside while a debitage *in situ* provided smaller flakes with various technologies including Levallois.

## Context of the Flake

The flake (G8 128) is a Levallois flake 60 mm long. The artefact, with the adhering cord fragment, was found *in situ* in level 4.2, 3 meters below the modern surface by the director of the excavations (M.-H. Moncel). Furthermore, the cord fragment was found on the inferior surface of the flake, meaning that the cord fragment entered the deposit contemporaneous with or before the flake. There is no evidence of a burrow or den or other disturbance to the sediment in the well-preserved stratigraphic sequence. Upon excavation, the artefact was immediately placed, unwashed, in a zip-style plastic bag where it remained until microscopic examination. This careful treatment of the artefact precludes further modern contamination.

## Results and Discussion

**Figure 2:**
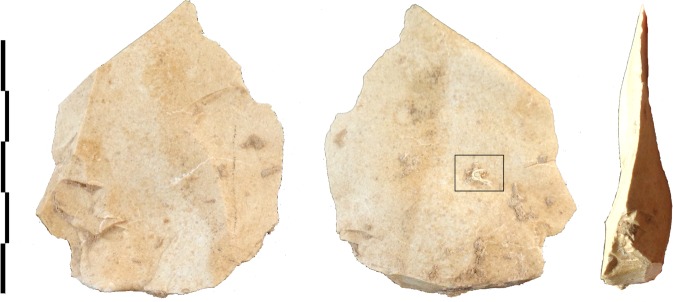
Levallois flake (G8 128 Level 4.2) with adhering cord fragment. (Photo by M.-H. Moncel).

**Figure 3:**
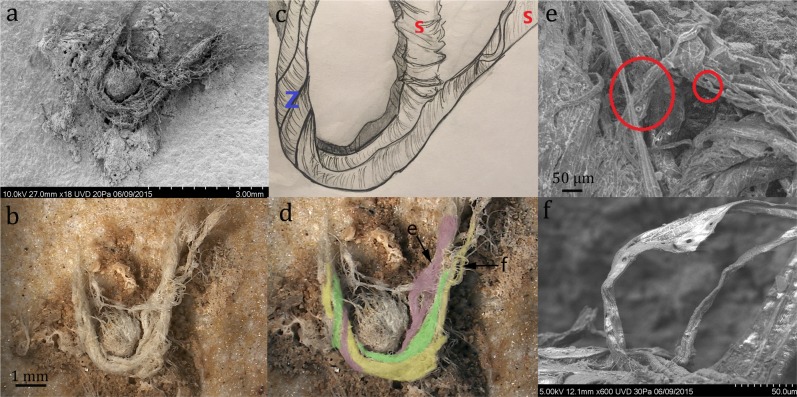
(**a**) SEM photo of cord fragment, (**b**) 3D Hirox photo of cord fragment, (**c**) schematic drawing illustrating s and Z twist; (**d**) enlarged Hirox photo with cord structure highlighted, arrows indicate location of photos e and f; (**e**) SEM photo of bordered pits (circled in red); (**f**) SEM photo of bordered pits. (Drawing by C. Kerfant; Hirox: C2RMF, N. Mélard).

**Figure 4:**
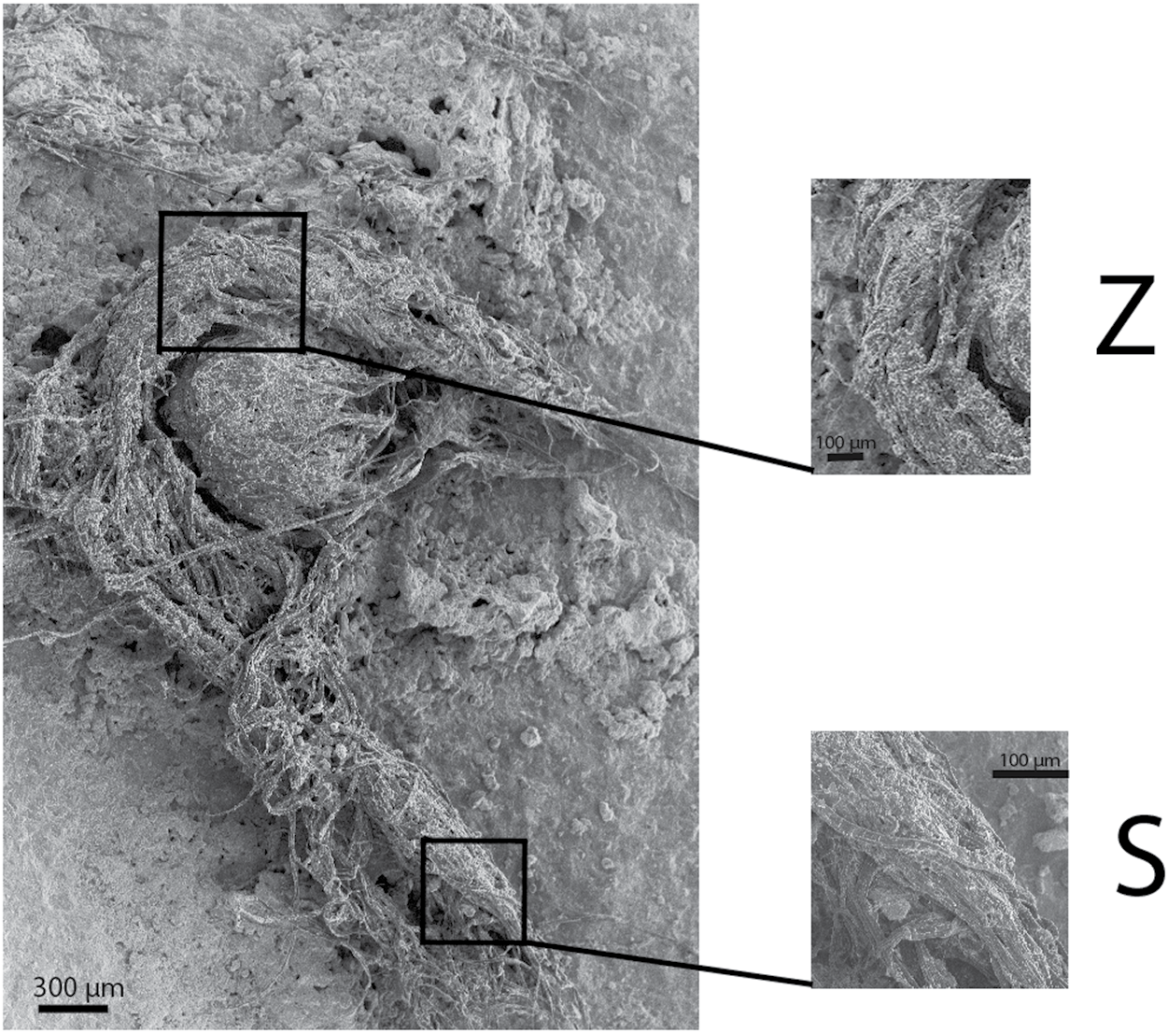
SEM photo of 3-ply cord. First closeup shows Z-twist of strands (image rotated 90° counter-clockwise for clarity); 2nd closeup shows S-twist of fibres within a single strand.

Samples of stone tools from the site are routinely screened with optical light microscopy. Previously, individual twisted plant fibres, some of which were multicellular, were reported on stone tools from Abri du Maras^6^. The authors suggested that these might be remnants of cordage, but the remains were too fragmentary to be conclusive. During further screening with reflected light microscopy, we discovered a fragment of string on a Levallois flake (sample G8 128) from Level 4.2 (Fig. 2). The flake was recovered *in situ* with the cord adhering to its inferior surface and was covered by sediment and breccia, demonstrating that the cord is at least contemporary with the deposition and burial of the flake and is therefore Middle Paleolithic in origin. The specimen was also imaged using an environmental SEM imaging platform of the National Museum of Natural History (MNHN, Paris) and a Hirox 2D/3D digital microscope at the Centre for Research and Restoration of the Museums of France (C2RMF, Paris). Examination of photomicrographs revealed 3 bundles of fibres with S-twist which were then plied together with a Z-twist to form a 3-ply cord^14^. The cord is approximately 6.2 mm in length and approximately 0.5 mm in width (Figs. 3, 4). The morphology of the cord fragment closely resembles replica cords produced in modern materials (see SI Fig. 1). Based on the presence of bordered pits^15^ with torus-margo membranes which are arranged in parallel lines, the fibres resemble gymnosperm (conifer) and come from the inner bark^16,17^. The torus is surrounded by a margo that controls the pressure in the conifer water transport system,; this mechanism is a strategy that distinguishes gymnosperm from angiosperm (flowering plants)^18,19^. Juniper, spruce, cedar, and pine bast have been used archaeologically and historically in the manufacture of cordage and textiles (see Supplemental Information). The presence of pine at the Abri du Maras is confirmed through palynological^20^ and charcoal analysis^12^. We also collected modern fibre samples from 18 materials that were present during the excavations and examined them microscopically. None of these matched the fibres from sample G8 128.

**Figure 5:**
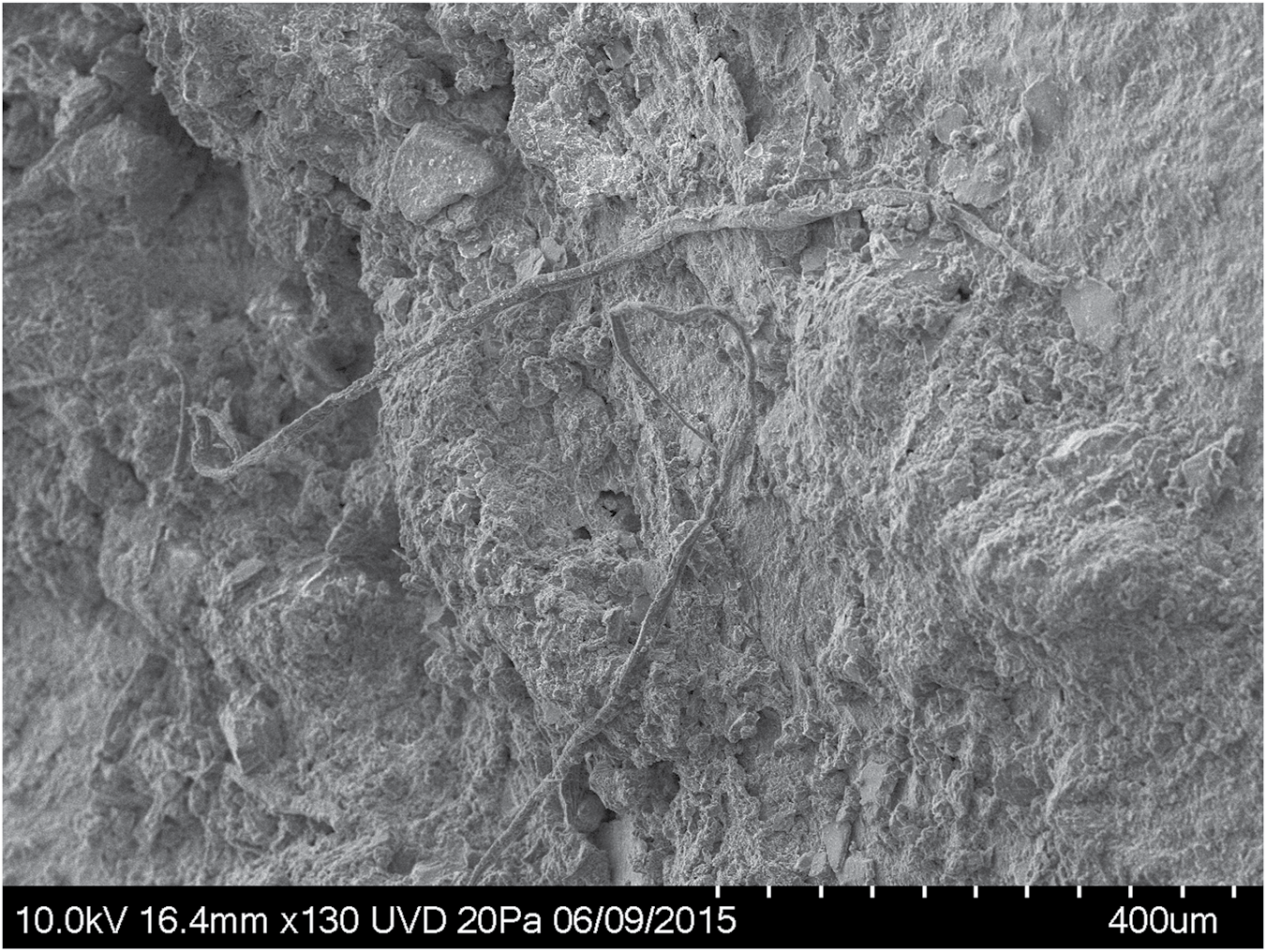
SEM photo of untwisted fibres on artefact L6 791.

**Figure 6:**
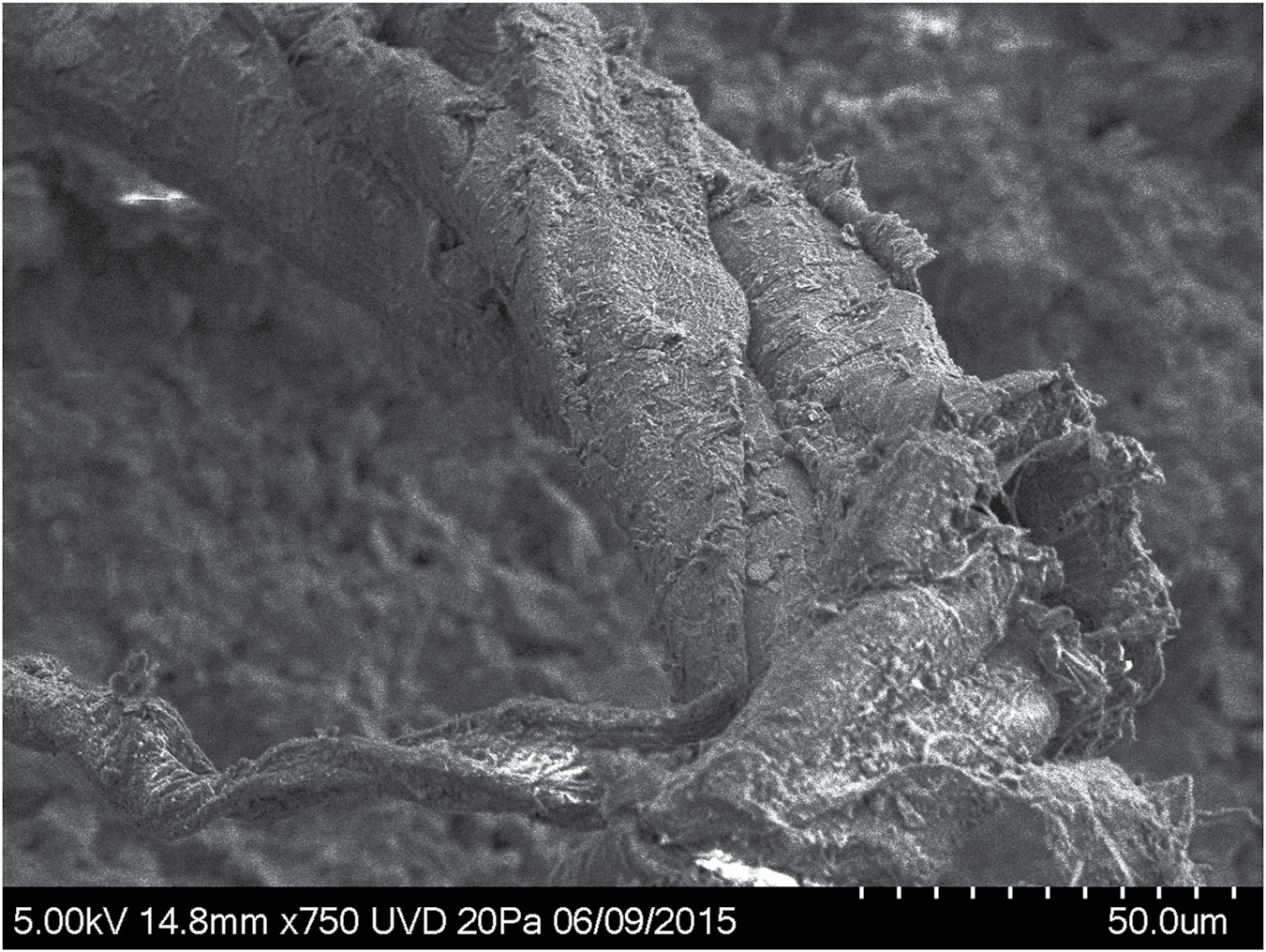
SEM photo of multiple fibres showing a Z twist on artefact L6 791.

**Figure 7:**
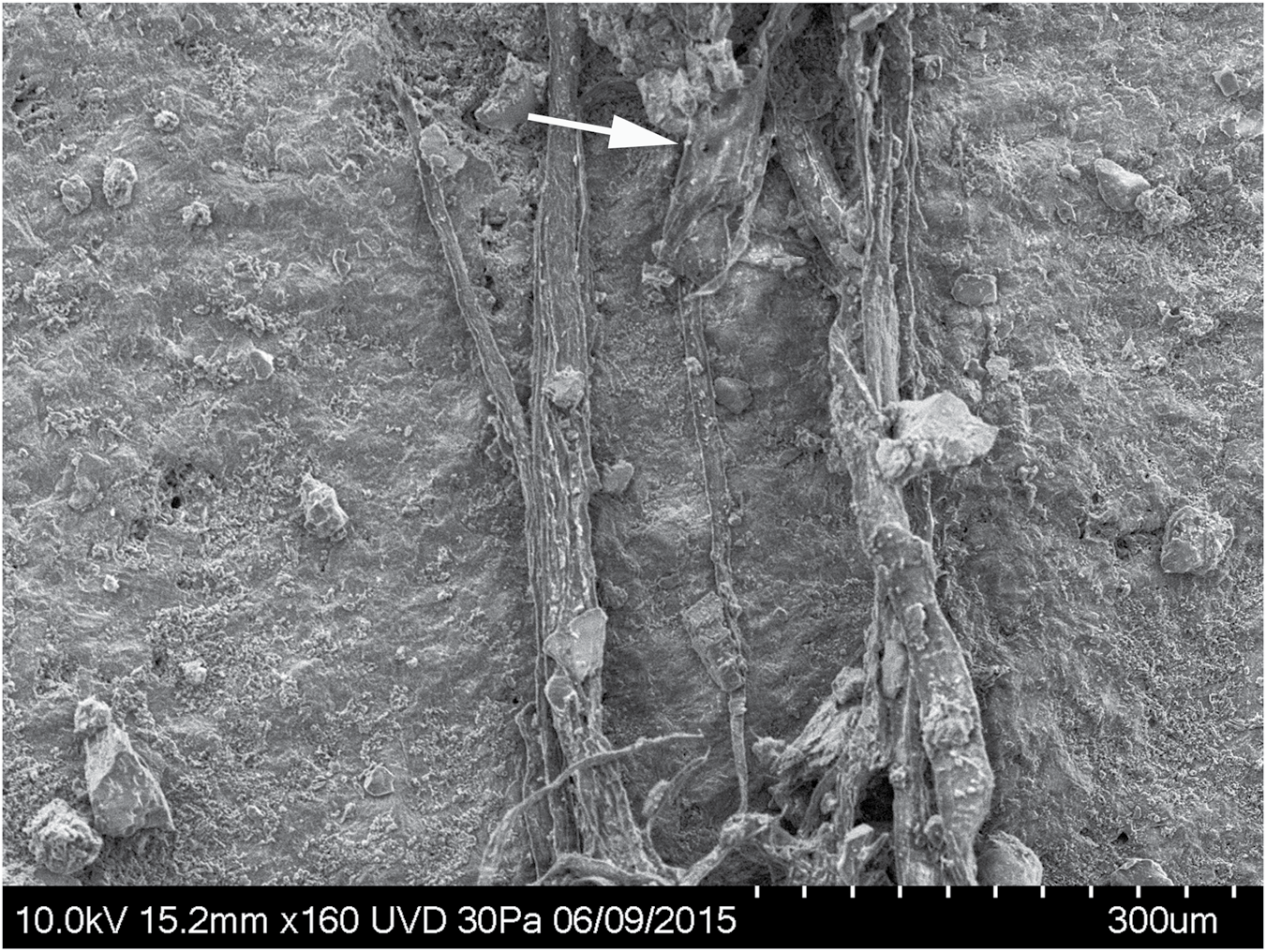
SEM photo of multiple untwisted fibres on artefact I6 333. Arrow indicates bordered pits suggesting a conifer origin for the fibres as with the cord fragment on G8 128.

In addition to the cord fragment described here and examples of twisted fibres illustrated in previously published photos^6^, a number of artefacts have plant/wood fibres adhering to their surfaces but do not exhibit sufficient twisting or plying to confidently identify them as remains of cordage. In some cases these show some twists while in other cases they do not. It is possible that these fibres are related to cordage or cordage manufacture, but, thus far, the sample on flake G8 128 is the only one to exhibit clear structure of a multiple ply cord. Figures 5–7 show fibres on artefacts L6 791 (Level 4.2) and I6 333 (Level 4.1). Both artefacts were found *in situ* and were handled in the same manner as artefact G8 128 and the cordage fragment (unwashed, placed in zip style plastic bags until microscopic analysis).

**Figure 8:**
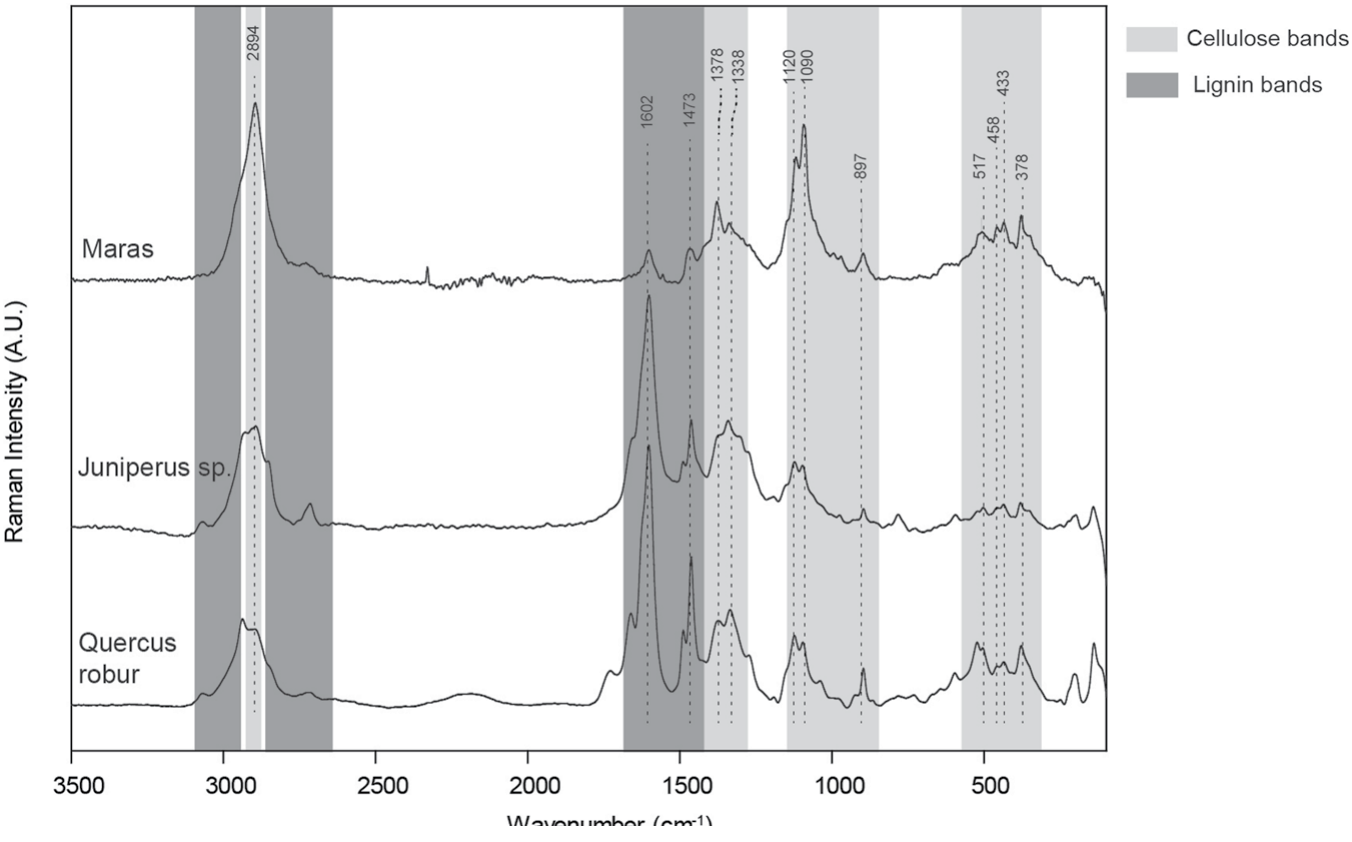
Representative spectrum obtained by FT-Raman spectroscopy on Maras fibres on artefact G8 128 compared to those acquired on modern juniper and modern oak.

We applied FT-Raman spectroscopy to the cord on sample G8 128 to determine the composition of the preserved fibres (Fig. 8). Raman spectrometry was the most suitable technique considering is high spatial resolution and its non-invasive application. This technique is becoming increasingly common in analysis of residues on stone tools^21,22^ and is used here to corroborate visual identification of plant residues.

Raman spectra analysis exhibits several bands characteristic of cellulose (1378, 1338, 1120, 1090, 897, 519 cm^−1^). The weaker presence of lignin is also attested to by the band at 1602 cm^−1 ^^23,24^. Several micro-analyses were performed at different locations on the cord sample and yielded the same spectral signature illustrated in Fig. 5. Analyses focused outside the cord sample did not exhibit an organic substance signature. These results confirm that the molecular nature of these residues is consistent with an interpretation of wood components. Spectra from modern *Quercus* and *Juniperus* are included as examples of hardwood and softwood respectively. Note that the peaks match, but that obviously the Maras sample has been degradated. The proportions of lignin and cellulose vary according to wood species and in particular between hardwood (angiosperms) and softwood (gymnosperms, including conifers). Holocellulose (cellulose and hemicellulose) is the main component of woods with the proportions varying between 65–80% for softwood and between 70–90% for hardwood, the remainder consisting mainly of lignin. Cellulose is typically more sensitive to degradation than lignin, with generally low cellulose content in archaeological samples^25^.

However, burial in an alkaline environment like carbonaceous bedrock can induce a preferential degradation of lignin and hemicellulose^26^. Aging could have thus modified the relative proportions of lignin and cellulose in the analyzed fibres and it is not possible to reconstruct the initial composition because of their alteration. These results highlight the exceptional preservation of this organic material with the conservation of the cellulose molecular structure. The localization of this material, in contact with a carbonaceous breccia, could have favored the deposits of calcite minerals protecting the fibres from further alterations. Previous work has also demonstrated that the flint at Abri du Maras is covered with a microscopic post-depositional film of chalcedony, which may also aid in preservation^27^.

The cord is not necessarily related to the use of the tool. Its presence on the inferior surface of the flake during excavation demonstrates that it was deposited before or contemporaneous with the flake. If it was contemporaneous with the deposition of the flake, it could have been wrapped around it as part of a haft or could even have been part of a net or bag. Previous analysis of impact fractures on artefacts from the site suggests the use of hafting and provide potential support for this possibility^6^. If it was deposited before the flake, it could represent a number of different items but nonetheless illustrates the use of fibre technology at the site.

At present, the earliest possible evidence of fibre technology are the shell beads from Cueva Anton with a minimum age of 115 ka^28^. Shell beads may have been strung or tied to clothing as personal ornamentation, although this could have been accomplished with sinew or a leather thong as well as cordage. Conard and Malina^29^ have recently posited that perforated ivory artefacts (*lochstäbe*) from Aurignacian sites in the Swabian Jura were used for spinning plant fibres for rope-making or textiles. Other early indirect evidence of fibre technology is impressions on fired clay from Gravettian sites in Moravia as early as 28 ka^3^. These impressions reveal weaving technology and the production of textiles. The complexity of the textiles suggests that they are part of a well-established tradition that began much earlier.

In terms of the actual preservation of fibre technology, the Upper Paleolithic waterlogged site of Ohalo II yielded three fragments of fibres with a Z twist approximately 19 ka^30^. Remnants of a 6-ply cord were found at Lascaux and date to approximately 17 ka^31^. The cord fragment from Abri du Maras is older still, dating to between 41 and 52 ka. Thus, it appears increasingly likely that fibre technology is much older than previously thought.

While it is clear that the cord from Abri du Maras demonstrates Neanderthals’ ability to manufacture cordage, it hints at a much larger fibre technology. Once the production of a twisted, plied cord has been accomplished it is possible to manufacture bags, mats, nets, fabric, baskets, structures, snares, and even watercraft^3,4,32^. The cord from Abri du Maras consists of fibres derived from the inner bark of gymnosperms, likely conifers. The fibrous layer of the inner bark is referred to as bast and eventually hardens to form bark. In order to make cordage, Neanderthals had extensive knowledge of the growth and seasonality of these trees. Bast fibres are easier to separate from the bark and the underlying wood in early spring as the sap begins to rise. The fibres increase in size and thickness as growth continues. The best times for harvesting bast fibres would be from early spring to early summer. Once bark is removed from the tree, beating can help separate the bast fibres from the bark. Additionally, retting the fibres by soaking in water aids in their separation and can soften and improve the quality of the bast. The bast must then be separated into strands and can be twisted into cordage^4^. In this case, three groups of fibres were separated and twisted clockwise (s-twist). Once twisted the strands were twined counterclockwise (Z-twist) to form a cord.

Ropes and baskets are central to a large number of human activities. They facilitate the transport and storage of foodstuffs, aid in the design of complex tools (hafts, fishing, navigation) or objects (art, decoration). The technological and artistic applications of twisted fibre technologies are vast. Once adopted, fibre technology would have been indispensable and would have been a part of everyday life. In reconstructing land use patterns, paleoanthropologists typically give priority to activities such as hunting and acquiring lithic raw material. Fibre acquisition, processing, and production may have also played an important role in scheduling daily and seasonal activities. String and rope manufacture are time intensive activities and large amounts of string are required for the production of carrying objects such as bags. In an ethnomathematical study of the Maya, Chahine^33^ found that a 1.3 foot Maguey bag required over 400 meters of cordage.

Thinking of the environment as including both natural and anthropogenic objects makes it possible to ask several questions about the choices made by cultural groups. Topography, climate, and distribution of plant and animal species are all key factors to consider. Plants play an important role not only in the material conception of objects but also in the formation of the thought of a culture, its representation of the world and its cosmogony^4^.

Overall, cordage manufacture has a complex *chaîne operatoire*. Although wooden artefacts are rare, other finds attest to Neanderthals detailed knowledge of trees. They chose boxwood for its density and used fire in the production of “digging sticks” at Poggetti Vecchi approximately 175 ka^2^. In the construction of the Schöningen spears, they decentered the point to increase strength^1^. Furthermore, Neanderthals were manufacturing birch bark tar in the Middle Pleistocene of Italy^34^ and at the sites of Konigsaue^35^ and Inden-Altdorf in Germany^36^. Based on this evidence, the utilization of bast fibres from trees is an obvious outcome of their intimate arboreal knowledge. While some have suggested that cordage manufacture may have been a gendered activity^37^, we feel our current evidence is inadequate to address that question.

Understanding archaeological finds in terms of taskscapes^38^, locating socially-situated tasks in the landscape, allows us to more fully appreciate the complexity of Neanderthal technology and social life. The production of cordage is complex and requires detailed knowledge of plants, seasonality, planning, retting, etc. Indeed, the production of cordage requires an understanding of mathematical concepts and general numeracy in the creation of sets of elements and pairs of numbers to create a structure^4,39^. Indeed, numerosity has been suggested as “one possible feral cognitive basis for abstraction and modern symbolic thinking”^40^^(p.205)^. Malafouris^41^ has suggested that a material instantiation of number concepts was necessary for the emergence of cognitive numerical ability. The production of cordage, with its use of pairs and sets, may represent one such instantiation. The production of the cord from Abri du Maras requires keeping track of multiple, sequential operations simultaneously. These are not just an iterative sequence of steps because each has to have access to the previous stages. The bast fibres are first s-twisted to form yarn, then the yarns z-twisted (in the opposite direction to prevent unravelling) to form a strand or cord^42^. Cordage production entails context sensitive operational memory to keep track of each operation. As the structure becomes more complex (multiple cords twisted to form a rope, ropes interlaced to form knots), it demonstrates an “infinite use of finite means” and requires a cognitive complexity similar to that required by human language^43,44^.

The cord fragment from Abri du Maras is the oldest direct evidence of fibre technology to date. Its production demonstrates a detailed ecological understanding of trees and how to transform them into entirely different functional substances. Fibre technology would have been an important part of everyday life and would have influenced seasonal scheduling and mobility. Furthermore, the production of cordage implies a cognitive understanding of numeracy and context sensitive operational memory. Given the ongoing revelations of Neanderthal art and technology^2,45,46^, it is difficult to see how we can regard Neanderthals as anything other than the cognitive equals of modern humans.

## Materials and Methods

Stone tools from the site of Maras are minimally handled and placed in a sealed plastic bag until they can be analyzed microscopically for the presence of possible residues. The initial screening was done via reflected light microscopy at magnifications of 20-475x using DinoLite digital microscopes. Potential residues were photographed with Dinocapture 2.0 software and their position recorded on a line drawing of the artefact. Analysis of flake G8 128 revealed twisted fibre bundles. This specimen was also viewed using an environmental scanning electron microscope (Hitachi SU 3500) at a variety of magnifications and was examined with a Hirox RH-2000 (MXB-5000REZ lens) at the 2D/3D imaging platform of the National Museum of Natural History (MNHN, Paris).

To further characterize the specimen, we used Fourier Transform Raman spectroscopy (FT-Raman) to non-invasively analyse the molecular composition of fibre residues. FT-Raman spectroscopy using a Near-Infrared excitation source at 1064 nm was chosen to limit the effect of fluorescence that can occur when analyzing organic matter. Analyses were performed on a Bruker RFS 100/S system from MONARIS lab based on a Nd-YAG laser source, a Michelson-type interferometer and a nitrogen-cooled germanium detector.

The FT-Raman spectrometer is coupled with a microscope allowing analyses with a spot size of about 15 µm using a 100x long working distance infrared objective. In order to avoid sample alteration during analysis, a laser source power of 500 mW was used corresponding to ~ 120 mW on the sample. The non-contact analysis was performed by positioning the artefact directly on the microscope stage, focusing the laser beam at the desired location for analysis. Spectra were recorded between 50 and 3500 cm^−1^ with a spectral resolution of 4 cm^−1^, and between 10,000 to 40,000 scans were accumulated in order to obtain an improved signal to noise ratio. Multiple micro-analyses on different areas of the cord yielded the same spectral signature. Analyses outside the cord sample did not yield spectra with an organic signature.

## Supplementary information

Supplementary Information.
