# Author Correction: Direct evidence of Neanderthal fibre technology and its cognitive and behavioral implications

**DOI:** 10.1038/s41598-020-65143-5

**Published:** 2020-05-13

**Authors:** B. L. Hardy, M.-H. Moncel, C. Kerfant, M. Lebon, L. Bellot-Gurlet, N. Mélard

**Affiliations:** 1 Department of Anthropology, Kenyon College, Gambier, Ohio USA; 2 Histoire Naturelle de l’Homme Préhistorique (HNHP), UMR 7194, Dept. Homme et Environnement du Muséum national d’Histoire Naturelle, CNRS, UPVD, Institut de Paleontologie Humaine-Musée de l’Homme, 75013 Paris, France; 3 Àrea de Prehistòria, Universitat Rovira i Virgili (URV), Av. Catalunya 35, 43002 Tarragona, Spain; 4 IPHES, Institut Català de Paleoecologia Humana i Evolució Social, Zona Educacional 4 Campus Sescelades URV (Edifici W3), 43007 Tarragona, Spain; 5 5 Sorbonne Université, CNRS, De la Molécule aux Nano-objets: Réactivité, Interactions et Spectroscopies (MONARIS), UMR 8233, 75005 Paris, France; 6 Centre de Recherche et de Restauration des Musées de France, C2MRF Palais du Louvre, Paris France

Correction to: *Scientific Reports* 10.1038/s41598-020-61839-w, published online 09 April 2020

This Article contains errors in Reference 46 which was incorrectly given as:

Sykes, R. W. In *Settlement, Society and Cognition in Human Evolution* (eds. Coward, F., Hosfield, R., Pope, M. & Wenban-Smith, F.) 117–137 (Cambridge, Cambridge Univ. Press, 2015).

The correct reference is listed below as ref. 1:
