# Supplementary material for: Direct evidence of Neanderthal fibre technology and its cognitive and behavioral implications
Source: Sci Rep. 2020 Apr 9;10:4889. doi: 10.1038/s41598-020-61839-w (PMC7145842; doi:10.1038/s41598-020-61839-w)
Supplement: Supplementary file 1 — Supplementary Information. [file 41598_2020_61839_MOESM1_ESM.pdf]

Direct evidence of Neanderthal fibre technology and its cognitive and behavioral implications

Authors: B. L. Hardy[1*], M.-H. Moncel[2], C. Kerfant[3,4], M. Lebon[2], L. Bellot-Gurlet[5], N. Mélard[6]

Supplementary Information

Modern Experimental Cordage

In order to understand the mechanics of cordage manufacture and its microscopic anatomy, the corresponding author (BH) maintains an extensive experimental database of materials for comparison with archaeological residues. Supplemental Figure 1 shows a 2 ply flax cord with morphology similar to the cordage fragment on Flake G8 128 from Abri du Maras. Supplemental Figure 1a shows bundles of s-twisted fibres which are then Z twisted to form a 2 ply cord. Supplemental Figure 1b shows a portion of the cord which is partially unravelled and resembles the condition seen in parts of Figure 3 and 4 in the article text.

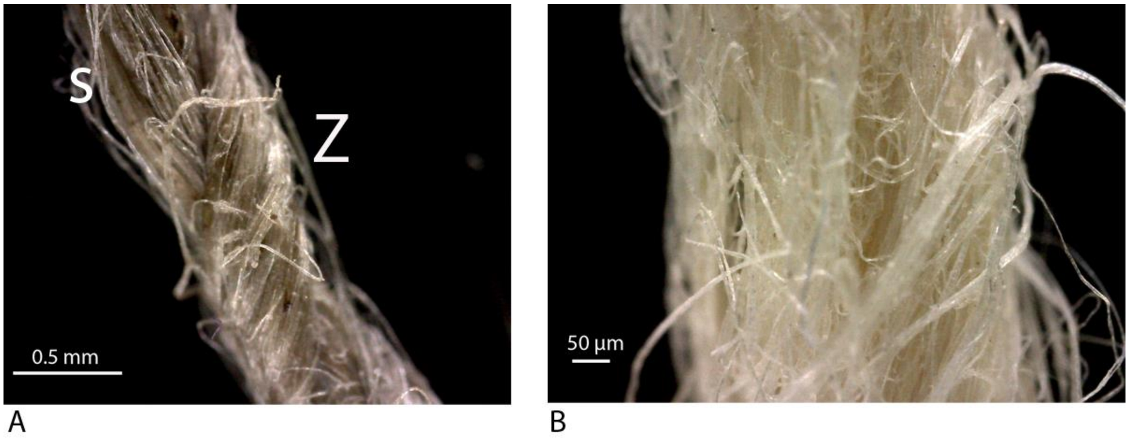

Figure 1: 2 ply experimental cord made of flax fibres. Photo A shows s twist of individual fibres in a strand and Z twist of strands to form a 2 ply cord. Photo B shows partially unraveled fibres where cord is not tightly twisted, similar to some parts of cord from Maras. Images taken under reflected light optical microscopy. These images are part of an extensive experimental database maintained by the corresponding author (BH).

Ethnographic and Archaeological Fibre Technology

Fibres derived from conifers (including spruce, cedar, pine, and juniper) have been used both archaeologically and historically in the production of cordage and other materials. A search

of the eHRAF (Human Relations Area Files) World Cultures database reveals a minimum of 18 different cultures in Europe and North America that used conifer bast and roots in the construction of a variety of material culture items. Among the Saami, spruce and pine ropes could last for 10-20 years[1]. Besides just the construction of cordage, other uses include seine nets[2], baskets and containers[3], watercraft[4], rainhats[3] and textile arts[5]. Vegetal technology was crucial for Tlingit and Kwakiutl of northwest North America who did not use pottery or metals. Red cedar roots were made into finely woven baskets, which were used such as containers, cooking tools, nets, plaits, or bags[6,7]. Pine needles can form a stringy material named "forest wool" that can be easily spun[8].

The use of conifers is also seen prior to historical times. Besides the evidence from Abri du Maras, a woody part of conifer bark have been extracted from the dental calculus of a Neanderthal individual of El Sidron Cave, Spain (c. 46 ka)[9], attesting to the use of pine. A much more recent well-preserved basket dated at 4000 BC in the Thorne River Estuary is made of *Picea* roots[10]. Ropes of several meters made of Juniper branches (*Juniperus communis*) were discovered on the Viking site of Toftanes (9th -10th centuries), Faroes islands[11,12]. Confier bast fibres are also suitable for the manufacture of clothing as evidenced by a painted armband (probably juniper) recovered from Lizard Man Village site in Arizona dating to approximately 1100 AD[13].

This brief review of the uses of conifer basts demonstrates their suitability for not only cordage, but a wide range other possible uses including boats, baskets, clothing and art. While we cannot say which, if any, of these items were being produced by Neanderthals, the basis for all of them is the twisting and plying of fibres. Since we see at Abri du Maras that Neanderthals were twisting and plying cord, the potential applications of this technology are vast.

Supplementary References

1. Itkonen, T. I., & Minn, E. K. *Lapps in Finland Up To 1945. Vol. 1.* (Werner Söderström Osakeyhtiö, 1948). http://ehrafworldcultures.yale.edu/document?id=ep04-002.
2. Osgood, C. *Ingalik Material Culture.* (HRAF Press, 1970). http://ehrafworldcultures.yale.edu/document?id=na08-001.
3. Storm, J. & Capoeman, P. *Land of the Quinault.* (Quinault Indian Nation, 1990). http://ehrafworldcultures.yale.edu/document?id=nr17-006.
4. Smith, H. *Ethnobotany of the Meskwaki Indians*. (Public Museum of Milwaukee, 1928). http://ehrafworldcultures.yale.edu/document?id=np05-005.
5. Murdock, G. *Haidas of British Columbia: Our Primitive Contemporaries*. (Macmilliam, 1934). http://ehrafworldcultures.yale.edu/document?id=ne09-003.
6. Paul, F. *Spruce Root Basketry of the Alaska Tlingit*. (Univ. Michigan, 1944).
7. Turner, N. & Bell, A. The ethnobotany of the Southern Kwakiutl Indians of British Columbia. *Econ. Bot.* **27**, 257-310 (1973).
8. Médard, F. L'acquisition des matières textiles d'origine végétale en Préhistoire : L'apport des données expérimentales et ethnographiques. *Les Nouvelles de l'Archéologie* **114**, 23-28 (2008).
9. Radini, A. et al. Neanderthals, trees and dental calculus: new evidence from El Sidrón. *Antiquity* **90**, 290-301 (2016).
10. Busby, S. *Spruce root basketry of the Haida and Tlingit*. (Univ. Washington Press, 2003).
11. Bending, J. et al. Toftanes. A Viking Age Farmstead in the Faroe Islands. *Acta Archaeol.* **84**, 5-239 (2013).

12. Arge, S. V. Viking Faroes: Settlement, Paleoeconomy, and Chronology. *J. N. Atl.* **7**, 1-17 (2014).

13. J. Whittaker, S. Kamp, Sinagua Painted Armbands. *Kiva* **58**, 177-187 (1992).
